# Prospective Study of Ambient Particulate Matter Exposure and Risk of Pulmonary Embolism in the Nurses’ Health Study Cohort

**DOI:** 10.1289/ehp.1408927

**Published:** 2015-05-08

**Authors:** Vivian C. Pun, Jaime E. Hart, Christopher Kabrhel, Carlos A. Camargo, Andrea A. Baccarelli, Francine Laden

**Affiliations:** 1 Jockey Club School of Public Health and Primary Care, The Chinese University of Hong Kong, Shatin, Hong Kong; 2 Department of Environmental Health, Harvard T.H. Chan School of Public Health, Boston, Massachusetts, USA; 3 Channing Division of Network Medicine, Department of Medicine, Brigham and Women’s Hospital and Harvard Medical School, Boston, Massachusetts, USA; 4 Department of Emergency Medicine, Massachusetts General Hospital and Harvard Medical School, Boston, Massachusetts, USA; 5 Department of Epidemiology, Harvard T.H. Chan School of Public Health, Boston, Massachusetts, USA

## Abstract

**Background:**

Pulmonary embolism (PE) is the most serious manifestation of venous thromboembolism and a leading cause of sudden death. Several studies have suggested associations of venous thromboembolism with short-term particulate matter (PM) exposure; evidence on long-term PM and traffic exposure is mixed.

**Objectives:**

We examined the association of long-term exposure to PM_2.5_, PM_2.5–10_, and PM_10_ (PM with diameter of ≤ 2.5, 2.5–10, and ≤ 10 μm) and distance to roadways with overall incident PE and with PE subtypes in a cohort of U.S. women.

**Methods:**

The study included 115,745 women from the Nurses’ Health Study, followed from 1992 through 2008. Incident PE cases were self-reported biennially. Nonidiopathic PE were cases for which the medical record revealed an underlying health condition related to PE (i.e., surgery, trauma, or malignancy); idiopathic PE were cases with no such history. We used spatiotemporal models combining spatial smoothing and geographic covariates to quantify exposure at residential addresses, and Cox proportional hazards models to calculate hazard ratios (HR) and 95% confidence intervals (CIs).

**Results:**

PM_2.5_ averaged over 1 month (HR = 1.22; 95% CI: 1.04, 1.44) or 12 months (HR = 1.17; 95% CI: 0.93, 1.48) was associated with incident PE, after adjusting for known risk factors and PM_2.5–10_. Equivalent analyses restricted to PE subtypes showed a positive association for PM_2.5_ with nonidiopathic PE, but not with idiopathic PE. We did not find evidence of an association between distance to roadways and PE risk.

**Conclusions:**

We provide evidence that PM in the prior 1 and 12 months is associated with PE risk. Our results also suggest that women with underlying health conditions may be more susceptible to PE after PM exposure.

**Citation:**

Pun VC, Hart JE, Kabrhel C, Camargo CA Jr, Baccarelli AA, Laden F. 2015. Prospective study of ambient particulate matter exposure and risk of pulmonary embolism in the Nurses’ Health Study cohort. Environ Health Perspect 123:1265–1270; http://dx.doi.org/10.1289/ehp.1408927

## Introduction

Exposure to particulate matter (PM) air pollution has been associated with increased short- and long-term morbidity and mortality from heart disease and stroke (Brook et al. 2004, 2010; Dockery et al. 1993; Hoek et al. 2013; Miller et al. 2007; Pope et al. 2002). One of the plausible mechanistic pathways mediating such effects is hypercoagulability and enhanced thrombosis (Baccarelli et al. 2007; Brook et al. 2004; Utell et al. 2002). Procoagulant abnormalities are a hallmark of venous thromboembolism (VTE), which is the third most common cardiovascular disease after acute myocardial infarction and stroke, with annual incidence of 1–2 per 1,000 persons in developed countries (Oger for the EPI-GETBO Study Group 2000; Previtali et al. 2011; Rosendaal 1999). VTE encompasses deep vein thrombosis (DVT) and pulmonary embolism (PE). DVT refers to the formation of one or more blood clots in one of the body’s large veins, most commonly in the lower limbs; PE, the most serious manifestation of VTE, typically occurs when a portion of the blood clot breaks off and travels to the heart, ultimately lodging in the arteries of the lungs, potentially leading to death and disability (Office of the Surgeon General 2008; Piazza and Goldhaber 2006). PE is a leading cause of sudden death (Kürkciyan et al. 2000). Studies have showed that the mortality rate for patients with PE is 2- to 4-fold higher than that for patients with DVT, and 10–20% of PE patients die within 3 months after the event (Goldhaber et al. 1999; Naess et al. 2007).

Until recently, little attention has been given to understanding the association between PM exposure and incident VTE or PE risk compared with the attention given to heart disease and stroke. A limited number of short- and long-term studies have provided some insight into a potential association, though the findings are conflicting (Baccarelli et al. 2008, 2009; Dales et al. 2010; Kan et al. 2011; Martinelli et al. 2012; Milojevic et al. 2014; Shih et al. 2011). Therefore, in this study, we used data from the Nurses’ Health Study (NHS), a large and well-defined prospective cohort, to examine the association between long-term particulate exposures, distance to roadways and the risk of incident PE.

## Methods

*Study population.* The NHS began in 1976 with 121,701 female registered nurses 30–55 years of age. These nurses lived in one of 11 states in the United States (California, Texas, Florida, Massachusetts, Pennsylvania, Ohio, New York, New Jersey, Michigan, Connecticut, and Maryland) at enrollment, but later relocated throughout the United States, with at least 10 nurses in each of the 48 contiguous states by 1992. The NHS is approved by the institutional review board of the Brigham and Women’s Hospital, and participants provided implied informed consent through completion of study questionnaires. For the present study, we included nurses who had at least a single residential address in the contiguous United States between 1992 and 2008 if they had not previously reported a PE and had information available on the exposures of interest.

*Outcome assessment.* Biennial questionnaires on risk factors and health outcomes have been mailed to nurses since 1976 (Colditz et al. 1997). Details of the case identification and confirmation have been reported elsewhere (Kabrhel et al. 2011). Briefly, on each questionnaire, nurses were asked to report whether they had received a physician diagnosis of PE during the previous 2 years. For nurses who reported PE diagnosis and had no history of malignancy, medical records were obtained and reviewed by study physicians blinded to exposure status. Imaging was considered diagnostic of PE if a ventilation/perfusion lung scan was read by a radiologist as high probability for PE, or if there was a filling defect on contrast-enhanced computed tomography of the pulmonary vasculature or on catheter-based pulmonary angiography. Idiopathic PE was defined by the absence of recent surgery, major trauma, or active malignancy listed in the medical record. A diagnosis of nonidiopathic PE was made based on the presence of either *a*) surgery or major trauma within 1 month of PE diagnosis as listed in the medical record, or *b*) history of active malignancy and self-reported physician-diagnosed PE. Given the severity of diagnosis, followed by extensive treatment, therapeutic monitoring, and clinical follow-up required of patients with VTE, PE cases are likely reported with high accuracy in our population of nurses with high health literacy. This is supported by our ongoing medical record review of PE cases, and by a validation study of 101 self-reported cases of DVT for whom medical records were available. We found that 95 (94%) cases were confirmed, 2 (2%) cases were probable, and only 4 (4%) cases were not confirmed. Unlike PE, DVT diagnosis has not been routinely tracked in the NHS; therefore, the present study focused only on PE cases.

*Exposure assessment.* Monthly predictions of particulate matter ≤ 10 (PM_10_), ≤ 2.5 (PM_2.5_), or between 2.5 and 10 μm (PM_2.5–10_) were available for all residential addresses from nationwide spatiotemporal models. Details of the model development process have been provided elsewhere (Paciorek et al. 2009; Yanosky et al. 2008, 2009, 2014). Briefly, the generalized additive mixed models (GAMM) incorporated monitoring data from the U.S. Environmental Protection Agency (EPA) Air Quality System (AQS) (U.S. EPA 2015a), the Visibility Information Exchange Web System (VIEWS 2010), the Interagency Monitoring of Protected Visual Environments (IMPROVE) network (IMPROVE 2013), Stacked Filter Unit (a predecessor to IMPROVE) (IMPROVE 2015), Clean Air Status and Trends (CASTNet) networks (U.S. EPA 2015b), and Harvard research studies (Spengler et al. 1996; Suh et al. 1997) along with a number of geographic information system (GIS)–derived and meteorological covariates and spatial smoothing to estimate monthly prediction surfaces from 1989 forward. Because PM_2.5_ monitoring data are lacking before 1999, separate GAMM models were constructed for 1988–1998 and 1999–2007 (Yanosky et al. 2014). The pre-1999 model added extinction coefficients and a simpler spatiotemporal structure to estimate the PM_2.5_ to PM_10_ ratio seasonally, whereas the post-1999 PM_2.5_ model included point-source PM_2.5_ emissions. The models were evaluated using cross-validation; they exhibited little bias (slopes of the regression between predicted and measured of 0.87, 0.77, and 0.76 for PM_2.5_, PM_10_, and PM_2.5–10_, respectively) and high precision (mean absolute errors of 1.81, 5.44, and 4.73 μg/m^3^ for PM_2.5_, PM_10_, and PM_2.5–10_, respectively) (Yanosky et al. 2014).

We used distance from each residence to the nearest road as a proxy for traffic-related air pollution exposure. Specifically, the distance (in meters, m) from each residential address to the closest U.S. Census Feature Class Code A1 (roads with limited access, typically interstates), A2 (major, noninterstate roads), and A3 (secondary roads, typically with more than two lanes) road segments from the ESRI StreetMap Pro 2007 file were determined using a GIS (ArcGIS, version 9.2; ESRI, Redlands, CA). Distance to the closest road was categorized as 0–49 m, 50–199 m, 200–499 m, or ≥ 500 m, based on the distribution of distance in the cohort and exposure studies showing exponential decay in a number of exposures with increasing distance from a road (Karner et al. 2010).

*Covariates.* To assess potential confounding, time-varying data from the biennial questionnaires were used for the following covariates chosen *a priori* based on previous associations with either PE or air pollution: age (months), calendar year, season, region of residence (Northeast, Midwest, South, West), body mass index (BMI), smoking status (never, former, or current), pack-years of smoking (number of packs/day multiplied by number of years of cigarette smoking), having a high-risk diet according to the Alternative Healthy Eating Index score (Chiuve et al. 2012), total caloric intake, physical activity [< 3, 3 to < 9, 9 to < 18, 18 to < 27, or ≥ 27 metabolic equivalent (MET), hours/week], parity (nulliparous, 1, 2–3, or ≥ 4 children), menopausal status and hormone use, nonaspirin nonsteroidal anti-inflammatory drug use, warfarin use, multivitamin use, hypertension, coronary heart disease (any history of angina, coronary artery stenosis, or myocardial infarction), and rheumatologic disease (any history of systemic lupus erythematosus or rheumatoid arthritis). Race (white or nonwhite) and spouse’s highest educational attainment (high school, college, or graduate school) obtained from the 1992 questionnaire were included. Medication and vitamin use, hypertension, coronary heart disease, and rheumatological disease were treated as dichotomous variables. Area-level socioeconomic status at the U.S. Census tract level was adjusted for using median household value and median family income (U.S. Census Bureau 2000).

*Statistical analysis.* Time-varying Cox proportional hazards models were used to assess the association of PE for each 10-μg/m^3^ increase in predicted PM_2.5_, PM_10_, and PM_2.5–10_ exposures (after assessing linearity with cubic regression splines) and categories of distance to road. All survival models were based on a monthly time scale and were used to estimate hazard ratios (HRs) and 95% confidence intervals (CIs). Person-months of follow-up time were calculated from baseline (30 June 1992) until PE diagnosis, the end of follow-up (30 June 2008), censoring (moving outside the geographic region of interest or loss to follow-up), or death. We estimated the effect of average particulate exposure in the prior 12 months, because previous research in this cohort has shown time period to be the most relevant exposure for cardiovascular outcomes (Puett et al. 2009). In separate models, we also considered average exposure in the prior 1 month, given that shorter-term exposures may be more relevant for PE (Dales et al. 2010; Martinelli et al. 2012; Office of the Surgeon General 2008). We examined PM_2.5_, PM_10_, and PM_2.5–10_ individually in single-pollutant models. In sensitivity analyses, we assessed the robustness of the associations by examining PM_2.5_ and PM_2.5–10_ simultaneously in a multi-pollutant model. Basic Cox models were stratified by age in months and adjusted for year (linear term), region of residence and season. Confounding from covariates was evaluated individually in separate models and also simultaneously in full multivariable models. In addition, we conducted models restricted to after 2000 (when direct monitoring information on PM_2.5_ became available) and models restricted to women who had not moved residence for at least 5 years. All statistical analyses were conducted using SAS version 9.3 software (SAS Institute Inc., Cary, NC).

## Results

A total of 115,745 nurses were available for analysis, and selected participant characteristics during follow-up are presented in Table 1. Throughout the follow-up period, most were never or former smokers, white, and had given birth to at least two children. Nearly half of the nurses reported < 9 MET hr/week of physical activity, and were postmenopausal with no or past HRT use. Mean (± SD) levels of PM_2.5_, PM_2.5–10_, and PM_10_ exposures in the previous 12 months were 12.6 ± 3.0, 20.8 ± 5.8, and 8.2 ± 4.2 μg/m^3^, respectively. Most had residential addresses located ≥ 200 m from the nearest road, and only 15% were < 50 m from the nearest road.

**Table 1:** Age-standardized characteristics of the Nurses’ Health Study population throughout the follow-up period 1992–2008.

Characteristic	Study population
No. of women	115,745
Age (years) (mean ± SD)	65.9 ± 8.3
White race/ethnicity (%)	96.8
BMI (kg/m^2^) (mean ± SD)	25.6 ± 7.4
Smoking status (%)
Never	43.8
Past	43.4
Current	12.6
Missing	0.2
Pack-years of smoking^*a*^ (mean ± SD)	24.4 ± 21.5
High-risk diet score (mean ± SD)	164.3 ± 100.4
Total caloric intake (kcal/day) (mean ± SD)	1515.1 ± 724.7
Physical activity (%)
< 3 MET hr/week	20.6
3 to < 9 MET hr/week	20.7
9 to < 18 MET hr/week	18.5
18 to < 27 MET hr/week	11.4
≥ 27 MET hr/week	19.3
Missing	9.4
Husband’s education attainment (%)
Less than high school	4.0
High school	25.8
Greater than high school	35.2
Missing	35.0
Parity (%)
Nulliparous	5.8
1	7.3
2–3	55.0
≥ 4	29.9
Missing	2.1
Menopausal status (%)
Premenopausal	5.1
Postmenopausal and no HRT use	22.2
Postmenopausal and past HRT use	28.8
Postmenopausal and estrogen replacement therapy use	14.0
Postmenopausal and estrogen–progesterone replacement therapy use	9.5
Missing	20.4
Comorbidities
Hypertension (%)	46.7
Coronary heart disease (%)	3.0
Rheumatologic disease (%)	3.8
Nonaspirin nonsteroidal anti-inflammatory drug use (%)	22.1
Warfarin use (%)	2.2
Multivitamin supplement use (%)	45.2
Median family income^*b*^ (US$1,000s) (mean ± SD)	63.7 ± 24.7
Median house value^*b*^ (US$10,000s) (mean ± SD)	17.1 ± 12.7
Predicted PM_2.5_ (μg/m^3^) (mean ± SD)^*c*^	12.6 ± 3.0
Interquartile range	4.1
Predicted PM_10_ (μg/m^3^) (mean ± SD)^*c*^	20.8 ± 5.8
Interquartile range	6.4
Predicted PM_2.5-10_ (μg/m^3^) (mean ± SD)^*c*^	8.2 ± 4.2
Interquartile range	4.6
Distance to road [m (%)]
0–49	15.1
50–199	27.6
200–499	32.1
≥ 500	25.1
Abbreviations: HRT, hormone replacement therapy; MET, metabolic equivalent. ^***a***^Among smokers. ^***b***^Estimated for census tract of residence using data from the U.S. Census Bureau (2000). ^***c***^For 12 months prior.	

Over 1,725,024 person-years of follow-up, we identified 1,091 total cases of incident PE; 323 (29.6%) were confirmed idiopathic, 526 (48.2%) were nonidiopathic (283 in subjects with malignancy, 243 associated with surgery or trauma), and the subtype could not be determined for 242 (22.2%) cases (Table 2). The associations for spatiotemporal model–predicted ambient average PM_2.5_, PM_2.5–10_, and PM_10_ in the previous 1 or 12 months with incident PE, and the idiopathic and nonidiopathic subtypes are presented in Table 2. In basic models adjusted for age, calendar time, season, and region of residence, each 10-μg/m^3^ increase in prior 1-month or 12-month average PM_2.5_, PM_10_, or PM_2.5–10_ was associated with an increased risk of incident PE, and most associations were statistically significant. However, these increased risks were mostly observed within the nonidiopathic subtype for both time periods.

**Table 2:** Hazard ratios (95% confidence intervals) for pulmonary embolism (PE)*^a^* and its subtypes with a 10-μg/m^3^ increase in averaged predicted PM exposure in the 1 or 12 months before diagnosis (1,725,024 total person-years), single-pollutant models, 1992–2008 (*n* = 115,745).

Pollutants	Incident PE (1,091 cases)		Idiopathic PE (323 cases)		Nonidiopathic PE (526 cases)^*b*^	
	Basic^*c*^	Multivariable adjusted^*d*^	Basic^*c*^	Multivariable adjusted^*d*^	Basic^*c*^	Multivariable adjusted^*d*^
1-month average
PM_2.5_	1.24 (1.06, 1.45)*	1.22 (1.05, 1.43)*	0.98 (0.73, 1.31)	0.98 (0.73, 1.30)	1.26 (1.01, 1.58)*	1.26 (1.00, 1.57)*
PM_10_	1.11 (1.01, 1.22)*	1.10 (1.00, 1.20)*	0.97 (0.82, 1.16)	0.97 (0.81, 1.15)	1.07 (0.94, 1.22)	1.06 (0.93, 1.21)
PM_2.5–10_	1.08 (0.94, 1.23)	1.06 (0.92, 1.21)	0.96 (0.74, 1.24)	0.95 (0.73, 1.23)	0.98 (0.80, 1.20)	0.97 (0.79, 1.19)
12‑month average
PM_2.5_	1.27 (1.02, 1.57)*	1.24 (1.00, 1.54)	1.04 (0.69, 1.54)	1.03 (0.69, 1.53)	1.22 (0.89, 1.65)	1.21 (0.88, 1.65)
PM_10_	1.18 (1.04 1.32)*	1.15 (1.02, 1.30)*	1.08 (0.87, 1.35)	1.07 (0.86, 1.33)	1.10 (0.93, 1.30)	1.09 (0.92, 1.29)
PM_2.5–10_	1.23 (1.03, 1.46)*	1.19 (1.00, 1.42)*	1.17 (0.85, 1.62)	1.16 (0.83, 1.60)	1.09 (0.84, 1.40)	1.07 (0.83, 1.38)
^***a***^Regression results for 242 (22.2%) of the 1,091 cases with unknown PE subtype are not shown. ^***b***^Nonidiopathic PE includes confirmed secondary PE, and PE and previous cancer. ^***c***^Adjusted for age in months, region, year, season. ^***d***^Adjusted for age in months, region, year, season, race/ethnicity, BMI (continuous), smoking status, pack-years, Alternate Healthy Eating Index score, total caloric intake, physical activity, husband’s education attainment, parity, menopausal status, hypertension, coronary heart disease, rheumatologic disease, nonaspirin nonsteroidal anti-inflammatory drug use, warfarin use, multivitamin supplement use, median family income in census tract of residence, and median house value in census tract of residence. *p < 0.05.						

After adding each potential time-varying confounder one at a time to the basic model, the relationships of PM exposures with PE did not change appreciably relative to the basic models (data not shown). Multivariable adjusted models included race, BMI, smoking status, pack-years, diet, total caloric intake, physical activity, husband’s education attainment, parity, menopausal status, hypertension, coronary heart disease, rheumatologic disease, nonaspirin nonsteroidal anti-inflammatory drug use, warfarin use, multivitamin supplement use, median family income in census tract of residence, and median house value in census tract of residence (Table 2). Results from multivariable models were similar to those from basic models (Table 2), as were results from models restricted to after 2000 or to women who had not moved for at least 5 years (data not shown).

Table 3 shows the sensitivity analyses where we considered exposures to PM_2.5_ and PM_2.5–10_ simultaneously. Most HRs for incident PE were modestly attenuated relative to HRs in the single size fraction models, except for the estimate of 10-μg/m^3^ increment in PM_2.5_ level averaged over the prior 1 month (HR = 1.22; 95% CI: 1.04, 1.44). Equivalent analyses restricted to specific PE subtypes showed that PM_2.5_ exposure averaged over the prior 1 and 12 months, adjusted for PM_2.5–10_, was associated with increased risk of nonidiopathic PE [1-month HR = 1.30 (95% CI: 1.03, 1.65); 12 month HR = 1.20 (95% CI: 0.86, 1.67)], but not with idiopathic PE. Although elevated incident PE risk was linked to PM_2.5–10_ exposure in single size fraction models, the inclusion of PM_2.5_ led to a decrease in HRs, and the associations were no longer statistically significant.

**Table 3:** Hazard ratios (95% confidence intervals) for pulmonary embolism (PE)*^a^* and its subtypes with a 10-μg/m^3^ increase in averaged predicted PM exposure in the 1 or 12 months before diagnosis (1,725,024 total person-years), multi-pollutant models, 1992–2008 (*n* = 115,745).

Pollutants	Incident PE (1,091 cases)		Idiopathic PE (323 cases)		Nonidiopathic PE (526 cases)^*b*^	
	Basic^*c*^	Multivariable adjusted^*d*^	Basic^*c*^	Multivariable adjusted^*d*^	Basic^*c*^	Multivariable adjusted^*d*^
1-month average
PM_2.5_	1.23 (1.05, 1.45)*	1.22 (1.04, 1.44)*	0.99 (0.74, 1.33)	0.99 (0.74, 1.33)	1.30 (1.03, 1.65)*	1.30 (1.03, 1.65)*
PM_2.5–10_	1.02 (0.88, 1.18)	1.00 (0.87, 1.16)	0.96 (0.73, 1.26)	0.95 (0.73, 1.25)	0.91 (0.74, 1.13)	0.90 (0.73, 1.12)
12‑month average
PM_2.5_	1.19 (0.94, 1.49)	1.17 (0.93, 1.48)	0.96 (0.63, 1.48)	0.96 (0.62, 1.47)	1.20 (0.86, 1.67)	1.20 (0.86, 1.67)
PM_2.5–10_	1.17 (0.97, 1.41)	1.14 (0.94, 1.37)	1.18 (0.84, 1.67)	1.17 (0.83, 1.66)	1.03 (0.78, 1.36)	1.01 (0.77, 1.34)
^***a***^Regression results for 242 (22.2%) of the 1,091 cases with unknown PE subtype are not shown. ^***b***^Nonidiopathic PE includes confirmed secondary PE, and PE and previous cancer. ^***c***^Adjusted for age in months, region, year, season. ^***d***^Adjusted for age in months, region, year, season, race/ethnicity, BMI (continuous), smoking status, pack-years, Alternate Healthy Eating Index score, total caloric intake, physical activity, husband’s education attainment, parity, menopausal status, hypertension, coronary heart disease, rheumatologic disease, nonaspirin nonsteroidal anti-inflammatory drug use, warfarin use, multivitamin supplement use, median family income in census tract of residence, and median house value in census tract of residence. *p < 0.05.						

Last, we did not find evidence of an association between distance to roadways and PE risk (Table 4). For nurses living within 49 m of roads compared with those living further away (≥ 500 m), the multivariable adjusted HRs for incident PE, idiopathic PE, and nonidiopathic PE were 1.02 (95% CI: 0.83, 1.25), 0.92 (95% CI: 0.63, 1.33), and 1.20 (95% CI: 0.90, 1.61), respectively.

**Table 4:** Hazard ratios (95% confidence intervals) for pulmonary embolism (PE)*^a^* and its subtypes with proximity to nearest road (1,483,727 total person-years), 1992–2008 (*n* = 108,337).

Exposure	Person-years	Incident PE (954 cases)			Idiopathic PE (283 cases)			Nonidiopathic PE (454 cases)^*b*^		
		Cases	Basic^*c*^	Multivariable adjusted^*d*^	Cases	Basic^*c*^	Multivariable adjusted^*d*^	Cases	Basic^*c*^	Multivariable adjusted^*d*^
Distance to road (m)
0–49	224,188	156	1.04 (0.85, 1.28)	1.02 (0.83, 1.25)	46	0.94 (0.65, 1.36)	0.92 (0.63, 1.33)	83	1.21 (0.91, 1.62)	1.20 (0.90, 1.61)
50–199	410,044	250	0.93 (0.78, 1.12)	0.92 (0.77, 1.10)	67	0.76 (0.55, 1.06)	0.75 (0.54, 1.04)	128	1.03 (0.80, 1.34)	1.03 (0.80, 1.34)
200–499	476,700	314	1.03 (0.86, 1.21)	1.01 (0.85, 1.20)	92	0.91 (0.67, 1.23)	0.89 (0.66, 1.21)	136	0.97 (0.75, 1.24)	0.97 (0.75, 1.25)
≥ 500	372,796	234	1.00 (referent)	1.00	78	1.00	1.00	107	1.00	1.00
^***a***^Regression results for 217 (22.7%) of the 954 cases with unknown PE subtype are not shown. ^***b***^Nonidiopathic PE includes confirmed secondary PE, and PE and previous cancer. ^***c***^Adjusted for age in months, region, year, season. ^***d***^Adjusted for age in months, region, year, season, race/ethnicity, BMI (continuous), smoking status, pack-years, Alternate Healthy Eating Index score, total caloric intake, physical activity, husband’s education attainment, parity, menopausal status, hypertension, coronary heart disease, rheumatologic disease, nonaspirin nonsteroidal anti-inflammatory drug use, warfarin use, multivitamin supplement use, median family income in census tract of residence, and median house value in census tract of residence.										

## Discussion

In the NHS cohort of U.S. women, we observed an increased risk of incident PE for each 10-μg/m^3^ increase in prior 1-month (HR = 1.22; 95% CI: 1.04, 1.44) and 12-month (HR = 1.17; 95% CI: 0.93, 1.48) average exposures to PM_2.5_ after controlling for known risk factors and PM_2.5–10_. Equivalent analyses limited to specific PE subtypes showed a statistically significantly positive association for PM_2.5_ with nonidiopathic PE, but not with idiopathic PE. We did not find evidence of an association between distance to roadways and incident PE risk.

To our knowledge, this is the first study to report positive associations of particulate exposures with incident PE risk in a North American population. To date, only three epidemiologic studies have examined the association of long-term exposure to PM with VTE risk, and there has been little consistency in outcomes or exposures across the studies. In the first studies to examine the impact of pollution on DVT, Baccarelli et al. (2008) observed that each 10-μg/m^3^ increase in PM_10_ during the prior year was associated with a 70% elevation in DVT risk in healthy subjects in the Lombardia region of Italy. The risk was smaller in women (40% increase) and limited to those who were not using oral contraceptive or hormone therapy at the time of diagnosis. Two prospective American cohorts, the Women’s Health Initiative (WHI) Hormone Therapy trials and the Atherosclerosis Risk in Communities (ARIC) Study, reported no association between measures of PM_10_ and PM_2.5_ exposures and VTE risk (Kan et al. 2011; Shih et al. 2011). Our finding on distance to roadways and incident PE generally agrees with another American study reporting a null association of traffic density and distance to roads with VTE risk (Kan et al. 2011), but is in contrast to an Italian case–control study that found an inverse association between distance to roads and risk of DVT (Baccarelli et al. 2009). A possible explanation for the discrepant findings is the different primary disease outcomes across studies. In the ARIC and WHI studies, the main outcome was idiopathic and nonidiopathic combined VTE (Kan et al. 2011; Shih et al. 2011), whereas the main outcome in the Italian studies was first incidence of lower limb DVT with or without symptomatic PE (Baccarelli et al. 2008, 2009). We focused our analysis on incident PE, which is the most serious manifestation of VTE. We found evidence of an association between incident PE and PM_2.5_, but not with distance to roadway, a proxy measure for traffic-related pollution exposure. This suggests that other, non-traffic PM_2.5_ pollution exposures (e.g., industrial emissions) may underlie the association of PE with PM_2.5_.

A strength of our study was the ability to estimate the relative risks of idiopathic and nonidiopathic PE subtypes separately in association with PM. We found that the PM_2.5_ exposure was associated mainly with nonidiopathic PE, and the association between PM_2.5–10_ and idiopathic PE, though consistently positive, was nonsignificant. This is contrary to our hypothesis that air pollution would be more strongly associated with idiopathic PE cases given the general concept that air pollution induces a state of hypercoagulability and enhanced thrombosis. Although the precise biological pathways involved remain to be determined, our findings suggest a more complicated mechanism: that long-term PM exposure may be additive or interact with preexisting health conditions (in this case, active malignancy, recent surgery, or trauma) to promote PE, but may not participate in determining PE among individuals if they are not in the setting of some other risk factors of PE. Such findings may contribute to collective evidence indicating that certain characteristics, such as life stage, genetic polymorphisms, preexisting diseases, and socioeconomic status, may increase the susceptibility of populations to PM-related health effects (Peled 2011; Sacks et al. 2011). Our ability to examine PE subtypes and the fact that we observed different associations by subtype may help explain the conflicting results in previous studies. If the ratio of idiopathic to nonidiopathic VTE varies between studies, heterogeneity in findings may arise. Other explanations for the differential associations observed by PE subtypes include differential random variability, biases between groups, and fewer idiopathic cases than nonidiopathic cases. Further investigation of the effects of air pollution exposures on PE subtypes is warranted.

In addition to long-term effects of PM exposure, Dales et al. (2010) estimated increased hospital admissions for VTE and PE with daily levels of PM_2.5_ in a time-series analysis in Santiago, Chile, whereas Martinelli et al. (2012) estimated increases in emergency hospital admissions for VTE and PE associated with same day elevations of PM_2.5–10_, but not with PM_2.5_, in Verona, Italy. Although the underlying mechanisms of how particulate pollution may influence VTE pathogenesis (Virchow’s Triad) remain to be determined, it has been suggested that short- and long-term PM exposure enhances thrombotic and coagulation potentials by increasing circulating histamine, inflammatory cytokines, and platelets, plasma levels of coagulation proteins, such as procoagulant factor VIII, von Willebrand factor, and fibrinogen, and shortening prothrombin time (Baccarelli et al. 2007; Brook et al. 2010; Esmon 2003; Nemmar et al. 2006; Rückerl et al. 2006; Utell et al. 2002).

Several limitations of our study warrant consideration. First, we did not have information on the incidence of overall DVT or VTE, and we were not able to distinguish subtype for 22% of the PE cases. This limits our ability to examine the impact of PM on these other previously examined outcomes, as well as the power to detect statistically significant associations in our subtype analyses. Also, although self-report of PE has been well validated in this population, it is possible that some cases were not reported, leading to an underestimate of the effects of air pollution on PE. Second, our exposure estimates are predicted ambient, not personal, levels of PM. We do not have information on indoor sources, building characteristics that would influence the penetration of ambient exposures, or the amount of time each woman spent at her residence. These factors would all lead to misclassification in exposure, and would likely bias the estimates of association towards the null. Third, because we derived PM_2.5–10_ estimates from those of PM_10_ and PM_2.5_, they are subjected to two sources of random error in measurements, which may lead to lower statistical power. However, Yanosky et al. (2009) demonstrated that long-term average PM_2.5–10_ was reasonably well estimated (cross-validation *R*^2^ = 0.63 and 0.65 for long-term post-1999 and pre-1999 PM_2.5–10_, respectively). Third, the GIS-based temporal spatial smoothing of the PM predictive models reduced variability relative to measured concentrations; however, this Berkson measurement error should not cause substantial bias toward the null (Gryparis et al. 2009). Findings from the current study may not be generalizable to nonwhite racial/ethnic groups, to men, to younger women, and to socioeconomically deprived populations. Last but not least, it is possible that our results from this observational study are attributable to confounding. Adjustment for a variety of known confounding variables did not eliminate the observed associations, though there remains the possibility of residual confounding or confounding by unmeasured covariates (e.g., long-distance travel, recent prolonged inactivity) and/or by additional pollutants.

Nonetheless, our study had several strengths, including the ascertainment of incident PE cases and our ability to subtype cases based on detailed medical record review. PE is a reliable diagnosis based on evidence from objective methods such as ventilation/perfusion lung scan; thus the chance of false-positive PE cases is slim. The application of GIS-based spatiotemporal statistical models allowed us to assign exposures specific to biennially updated residential addresses for the entire follow-up period with little bias and high precision relative to other modeling approaches (Yanosky et al. 2008, 2009). Missing exposure data were minimized because of the use of our highly specific GIS-based spatial smoothing model. The availability of updated information on individual-level covariate status (e.g., smoking) throughout the follow-up period, enabled us to take into account possible confounding effects.

## Conclusion

We reported evidence of an association between exposure to PM_2.5_ and elevated risk of PE among women in the United States. By contrast, distance to road was not a risk factor for incident PE. Our findings suggest that women with underlying health conditions may be more susceptible to increased PE risk after PM exposure.
